# Genome-Wide Association Mapping of Resistance to *Exserohilum* Spike Blight in Wheat

**DOI:** 10.3390/plants15152351

**Published:** 2026-07-30

**Authors:** Tulasi Korra, Ram Chandra, Srushtideep Angidi, Uday Kumar Thera, Perumal Thirunarayanan, William Underwood

**Affiliations:** 1 Department of Mycology and Plant Pathology, Institute of Agriculture Sciences, Banaras Hindu University, Varanasi 221005, UP, India; rchandra@bhu.ac.in (R.C.); thirunarayanan.p1@bhu.ac.in (P.T.); 2 Department of Plant Pathology, Microbiology, and Biotechnology, North Dakota State University, Fargo, ND 58102, USA; srushtideep.angidi@ndsu.edu; 3 Department of Plant Science and Landscape Architecture, University of Maryland, College Park, MD 20740, USA; uthera@umd.edu; 4 USDA—ARS Sunflower Improvement Research Unit, Edward T. Schafer Agricultural Research Center, Fargo, ND 58102, USA

**Keywords:** wheat, *Exserohilum rostratum*, GWAS, quantitative resistance, marker–trait association

## Abstract

*Exserohilum* spike blight, caused by *Exserohilum rostratum,* is an emerging constraint in wheat production, and improving host resistance is a sustainable strategy because the disease is governed largely by quantitative, environment-responsive loci rather than single major genes. Identifying genomic regions and biological pathways underlying quantitative resistance is therefore essential for developing durable resistant varieties. In this study, the Wheat Association Mapping Initiative spring wheat panel (*n* = 289) was evaluated across two contrasting Indian agroclimatic zones over two seasons. Resistance was quantified using two traits: incubation period (IP) and area under the disease progress curve (AUDPC). Significant genotype, environment, and genotype × environment interaction effects were observed for both traits (*p* < 0.001). IP and AUDPC were weakly correlated (r = −0.18 to 0.16), indicating that these traits represent partially distinct resistance components. Genome-wide association mapping identified 25 marker–trait associations, 13 for AUDPC and 12 for IP, with most associations showing environment dependence. Putative candidate genes highlighted defense-relevant loci, including an LRR receptor-like kinase and a WRKY transcription factor for IP, and an RGA2-like resistance gene and PHLOEM UNLOADING MODULATOR-like gene for AUDPC. These findings provide the first GWAS-based framework for *E. rostratum* resistance in wheat, prioritizing loci for validation and marker deployment.

## 1. Introduction

Wheat (*Triticum aestivum* L.) is one of the most extensively cultivated cereal crops worldwide and represents a cornerstone of global food security. It contributes nearly one-fifth of the total caloric intake consumed by humans and is cultivated annually on more than 200 million hectares, with global production exceeding 750 million tons (http://www.fao.org, accessed on 28 January 2026). Due to its economic and nutritional importance, maintaining stable wheat production is crucial for sustaining food supplies and agricultural livelihoods.

Wheat is attacked by numerous fungal diseases that reduce yield and grain quality across diverse production environments. Among these pathogens, *E. rostratum* (Sacc.) Drechsler is a necrotrophic species primarily associated with grasses and cereals and is known to cause spike blight in wheat [1]. Taxonomically, *E. rostratum* is a phytopathogenic fungus belonging to the family Pleosporaceae (order Pleosporales, class Dothideomycetes, phylum Ascomycota, kingdom Fungi). It is morphologically characterized by darkly pigmented, distoseptate conidia with a distinctly protruding hilum—a diagnostic feature that distinguishes it from closely related genera such as *Bipolaris* [2,3]. According to MycoBank (MycoBank #314059, 2026), the genus currently comprises 38 accepted species, although more than 700 species were historically classified broadly under *Helminthosporium* before subsequent taxonomic revisions [4]. At the molecular level, *E. rostratum* can be identified through morphological characterization and multilocus sequencing of the ITS, GAPDH, TEF1-α, SSU, LSU, RBP-2, and β-tubulin genes, with ITS-rDNA and GAPDH sequence analysis providing robust resolution [5].

This pathogen is widely distributed throughout the world. It has been reported across several geographical regions, including South Asia (India, China, Sri Lanka, Bangladesh, and Pakistan), Africa, North America, Central and South America, Europe, and Oceania. The pathogen can survive between cropping seasons through infected crop residues, seeds, alternate hosts, and persistent conidia in soil, which facilitates its long-term persistence in wheat-growing agroecosystems [6]. Under favorable warm and humid conditions, conidia are dispersed by wind and rain splash to initiate primary infections, followed by secondary spread through newly produced conidia [7]. Furthermore, the sexual stage, *Setosphaeria rostrata*, develops through mating between compatible MAT1-1 and MAT1-2 isolates, resulting in pseudothecia and ascospores [8]. This sexual cycle contributes significantly to genetic recombination, pathogen survival, and population diversity. Genetic diversity analyses underscore this variability; for instance, in rice-growing areas of Burkina Faso, GBS-based analysis revealed moderate nucleotide diversity ($\pi =4.8\times 10^{-4}$) in *E. rostratum*, while population structure analysis showed that 94% of the genetic variation was present within pathogenic populations [9]. Previous studies have demonstrated that *E. rostratum* can colonize wheat tissues and disseminate through flower–seed–seedling transmission, highlighting its epidemiological relevance in wheat production systems [10]. Spike blight is a major seed-borne disease that represents a substantial constraint to wheat production, particularly in warm and humid agroecological regions where black spot pathogens are prevalent [11]. Spike blight indirectly reduces photosynthetically active leaf areas during critical growth stages, resulting in impaired grain filling and yield losses [12]. Because quantitative, environment-influenced responses typically govern resistance to such diseases, long-term reliance on chemical control is limited, making the development of genetically resistant cultivars one of the most effective and sustainable management strategies [13,14].

Disease development caused by *E. rostratum* is influenced by environmental conditions and host–pathogen interactions, and considerable variation in pathogenicity has been observed among fungal isolates [15]. Such variability suggests that virulence in *E. rostratum* is likely a complex quantitative trait governed by multiple genetic factors rather than a single major-effect gene. Although the survival biology and transmission pathways of this pathogen have been characterized, the genetic basis underlying wheat resistance to *E. rostratum* spike blight remains largely unexplored.

Genome-wide association studies (GWAS) provide an effective framework for dissecting the genetic architecture of complex, quantitative traits by exploiting historical recombination in diverse germplasm panels [16]. The Wheat Association Mapping Initiative (WAMI) panel, comprising elite spring wheat lines, was specifically designed to mitigate the confounding effects of phenology and plant height, and has been successfully utilized to identify genomic regions associated with agronomic and disease-related traits in wheat. GWAS has been widely applied to investigate resistance to several major wheat diseases, including *Fusarium* head blight, spot blotch, and *Septoria tritici* blotch [17,18]. However, wheat genomic regions associated with resistance to *E. rostratum* have not yet been characterized using GWAS.

Therefore, the objectives of the present study were to characterize resistance to *E. rostratum* in the WAMI panel using two complementary resistance-related traits: area under the disease progress curve (AUDPC) and incubation period (IP), to perform GWAS to identify associated genomic regions and prioritize candidate genes underlying resistance. The results provide a foundation for marker development, functional validation, and deployment of resistance loci in wheat improvement programs.

## 2. Results

### 2.1. Phenotypic Distribution of Disease-Related Traits

Field evaluation of the elite WAMI spring wheat panel across four environments (BHU–Varanasi and UAS–Dharwad over two seasons) revealed substantial phenotypic variation for both incubation period (IP) and area under the disease progress curve (AUDPC), indicating that the panel contains diverse levels of adult-plant response to *E. rostratum*. The combined ANOVA supported these observations by showing that genotype (G), environment (E), and the G × E interaction were all highly significant (*p* < 0.001) for both traits (Table 1). Significant genotype effects confirm that the elite germplasm differed genetically in terms of resistance components, while strong environmental effects demonstrate pronounced site-year dependence in disease expression. Importantly, the significant G × E interaction (*p* < 0.001) indicates that genotype performance was not fully consistent across environments, reinforcing the value of multi-environment phenotyping for identifying sources of resistance. 

**Figure 1:**
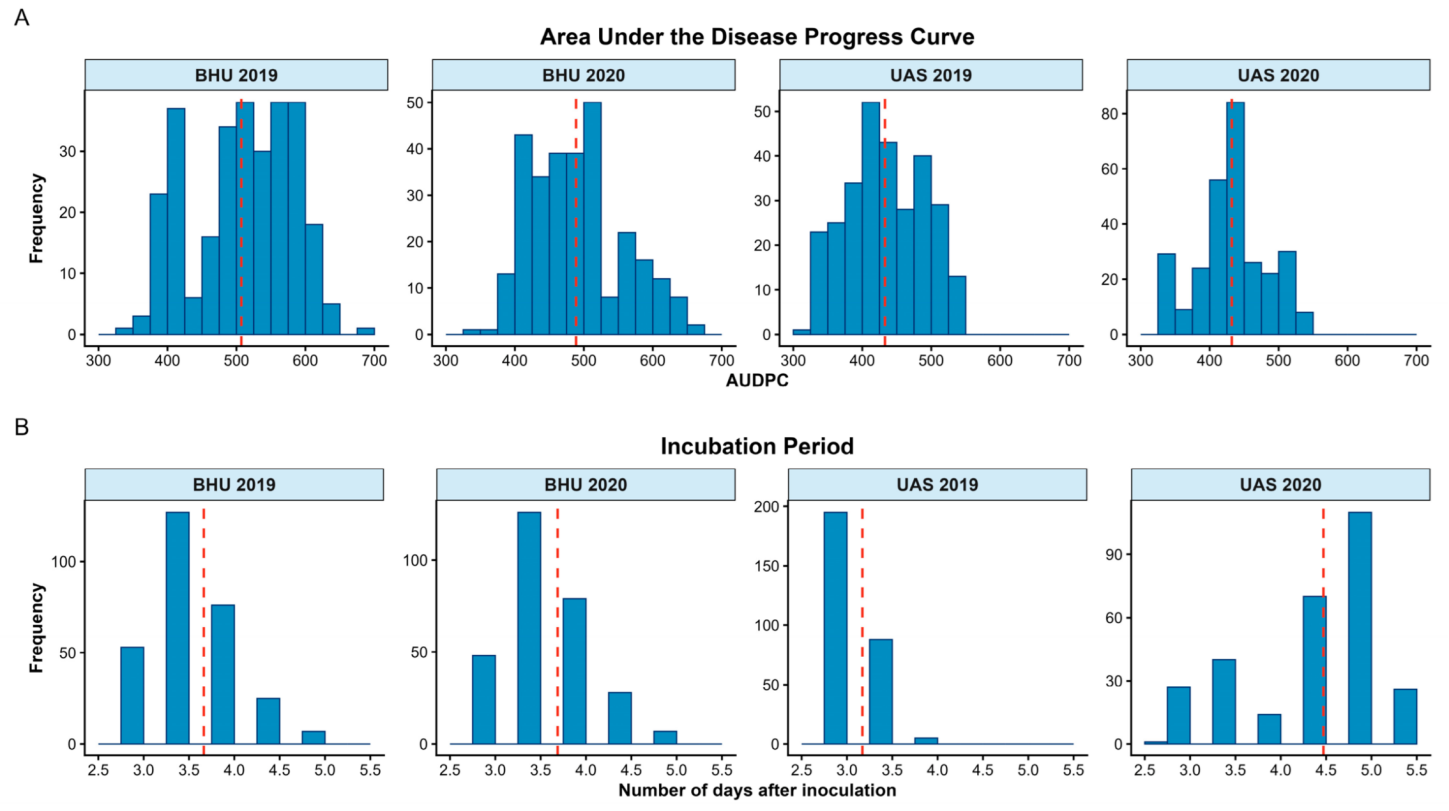
Frequency distributions of area under the disease progress curve (AUDPC) and incubation period (IP) for *Exserohilum* spike blight resistance in the WAMI wheat panel evaluated across four environments: BHU 2019, BHU 2020, UAS 2019, and UAS 2020. (**A**) Distribution of AUDPC values. (**B**) Distribution of IP values expressed as days after inoculation. Dashed red vertical lines indicate the mean value for each environment. The broad distributions observed for both traits indicate substantial quantitative variation across environments.

Across environments, IP ranged from 3.0 to 5.0 days at BHU and from 2.5 to 5.5 days at UAS (Figure 1). Mean IP values were similar across seasons at BHU (3.66 days in 2019 and 3.69 days in 2020). In contrast, UAS exhibited greater seasonal variation, with a shorter mean IP in 2019 (3.17 days) and a markedly longer IP in 2020 (4.47 days). AUDPC values ranged from 350.0 to 689.5 at BHU and from 315.0 to 549.5 at UAS. Mean AUDPC was higher at BHU (507.10 in 2019; 488.71 in 2020) than at UAS (432.91 in 2019; 432.06 in 2020), indicating greater disease pressure in the Indo-Gangetic Plains environments.

**Figure 2:**
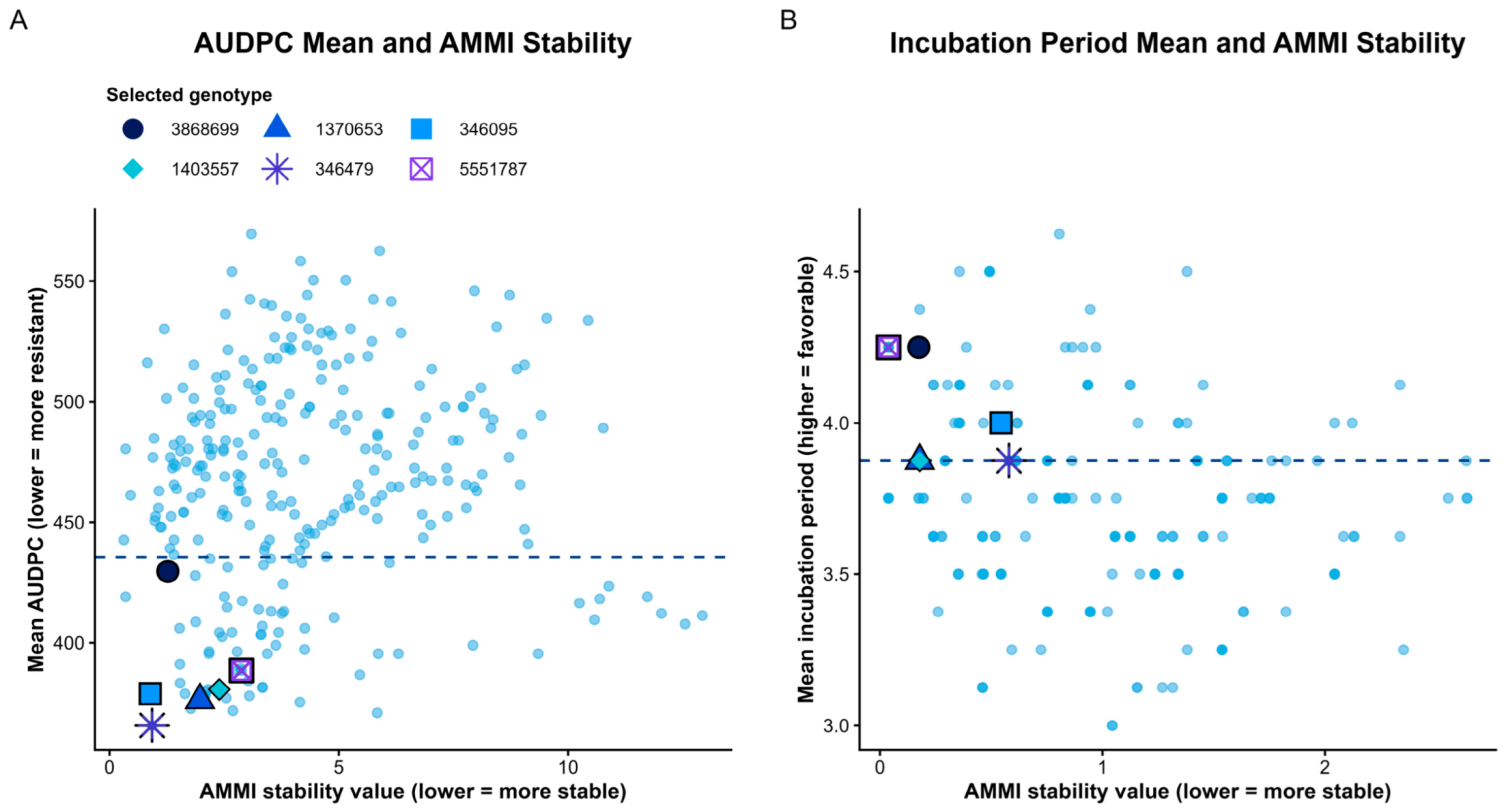
Relationship between mean disease response and AMMI stability value for wheat genotypes evaluated across four environments. (**A**) Mean area under the disease progress curve (AUDPC) plotted against AUDPC AMMI stability value. Lower AUDPC and lower ASV indicate greater resistance and stability, respectively. (**B**) Mean incubation period plotted against incubation-period ASV. A longer incubation period and lower ASV indicate a more favorable and stable response. Dashed horizontal lines indicate the lower-quartile AUDPC threshold and upper-quartile incubation-period threshold used to identify phenotypically favorable genotypes. Colored symbols indicate the six highest-ranked genotypes based on their combined AUDPC and IP stability ranks; the remaining genotypes are shown as light-blue circles.

Genotype stability across the four environments was evaluated using the AMMI stability value (ASV), with lower ASVs indicating greater stability. Twenty-six genotypes had mean AUDPC values within the lowest quartile and mean incubation-period values within the highest quartile (Supplementary Table S1). Among these, genotypes 3868699, 1370653, 346095, 1403557, 346479, and 5551787 formed the highest-ranked group based on their combined AUDPC and IP stability ranks (Table 2; Figure 2). Genotype 346479 had the lowest mean AUDPC within this group, whereas genotypes 3868699 and 5551787 had the longest mean incubation periods. Genotype 346095 had the lowest AUDPC ASV, while genotype 5551787 had the lowest IP ASV. These genotypes, therefore, showed favorable combinations of disease response and stability across the tested environments.

### 2.2. Correlation Between Incubation Period and Disease Severity

**Figure 3:**
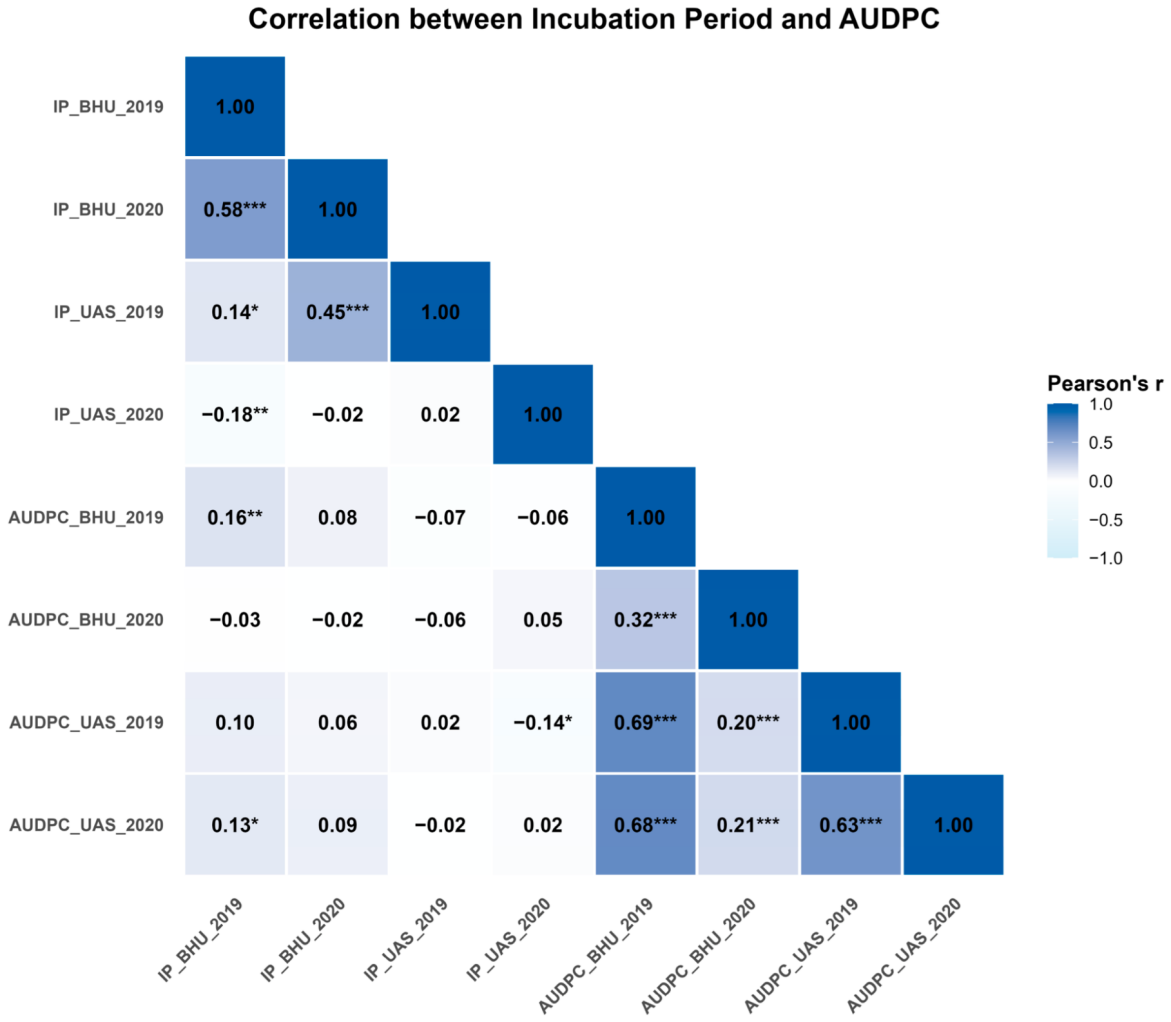
Correlation heatmap showing Pearson correlation coefficients between incubation period (IP) and area under the disease progress curve (AUDPC) across environments at BHU and UAS. AUDPC values showed moderate positive correlations across environments, whereas correlations between IP and AUDPC were generally weak and close to zero. Asterisks denote statistical significance (* *p* < 0.05, ** *p* < 0.01, *** *p* < 0.001).

Pearson correlation analysis was used to examine relationships among incubation period (IP) and area under the disease progress curve (AUDPC) across environments (Figure 3). AUDPC showed moderate-to-strong positive correlations across site-years, indicating a relatively consistent ranking of genotypes in overall disease development. For example, AUDPC at BHU-2019 was positively correlated with AUDPC at UAS-2019 (r = 0.69) and UAS-2020 (r = 0.68). Additionally, AUDPC at UAS-2019 and UAS-2020 were also positively correlated (r = 0.63).

Incubation period displayed moderate positive correlations across years within locations, with IP at BHU-2019 and BHU-2020 correlated at r = 0.58, and IP at UAS-2019 and UAS-2020 correlated at r = 0.45. In contrast, correlations between IP and AUDPC were generally weak and inconsistent across environments (r ranging from −0.18 to 0.16), suggesting that symptom onset timing and cumulative disease development capture largely distinct components of the host response. Accordingly, IP and AUDPC were treated as separate traits in subsequent genome-wide association analyses.

### 2.3. Marker–Trait Associations for IP and AUDPC

**Figure 4:**
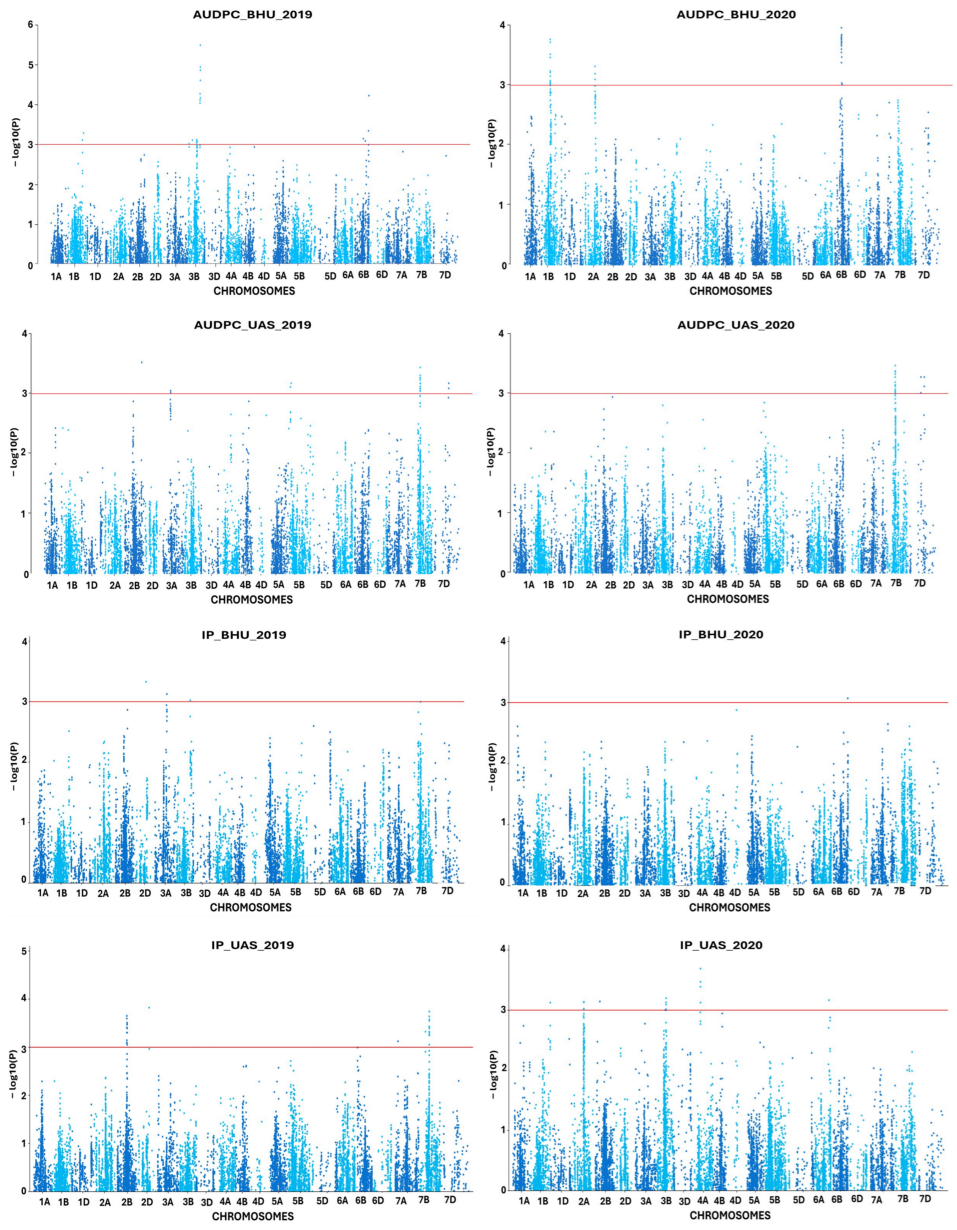
Manhattan plots showing genome-wide association results for incubation period (IP) and area under the disease progress curve (AUDPC) across environments using the FarmCPU model. The horizontal solid line indicates the significance threshold of −log_10_(*p*) ≥ 3.

Quantile–quantile (QQ) plots were used to evaluate model performance, and the Fixed and Random Model Circulating Probability Unification (FarmCPU) model, implemented in GAPIT, was selected because it provided the best balance between controlling spurious associations and retaining power to detect significant hits (Supplementary Figure S1). Using a reporting threshold of −log_10_(*p*) ≥ 3.0, we identified 23 significant marker–trait associations (MTAs) across environments (Table 3; Figure 4), including 13 MTAs for AUDPC and 10 MTAs for incubation period (IP).

Across the four site years, no single MTA was consistently detected in all environments, indicating that most association signals were environment-dependent. For AUDPC, MTAs were distributed across chromosomes 1B, 2A, 2B, 3A, 3B, 5B, 6B, 7B, and 7D. At BHU, associations were identified on 1B, 2A, 3B, and 6B, whereas at UAS, MTAs occurred on 2B, 3A, 5B, 7B, and 7D. Notably, Tdurum_contig43589_340_dup2 on chromosome 7D (position 141 cM) was detected in both seasons at UAS, representing the most repeatable AUDPC association within a location. In addition, a recurrent MTA was observed on chromosome 7B (position 73 cM) at UAS across seasons, although represented by different marker identifiers, suggesting a potentially stable genomic region that influences disease development under southern Indian conditions. For the incubation period, significant MTAs were detected on chromosomes 1B, 2A, 2B, 3A, 3B, 4A, 6A, 6B, 7A, and 7B, with the majority of MTAs identified under UAS conditions. No IP-associated SNP was consistently detected across years or locations, suggesting that genetic control of symptom onset may be more environment-sensitive than cumulative disease progression.

Several chromosomes harbored MTAs for both IP and AUDPC, including 1B, 2A, 2B, 3B, 6B, and 7B, indicating partial overlap in genomic regions contributing to different components of resistance. However, trait-specific signals were also observed, such as AUDPC associations on 5B and 7D and IP associations on 4A, 6A, and 7A, supporting the interpretation that IP and AUDPC capture related but distinct components of the host response to *E. rostratum*.

### 2.4. Putative Candidate Genes

Genes located within ±500 kb of each significant SNP were surveyed using the IWGSC Chinese Spring RefSeq v2.1 annotation. The complete gene lists and functional annotations for all associated intervals are provided in Supplementary Table S2. Putative candidates highlighted in the main text were selected based on combined evidence from marker proximity, predicted gene function, protein domain or orthology information, and previously reported roles of related genes in plant defense and stress responses.

Across traits, the annotated regions were a combination of defense signaling, regulatory control, and broader host physiological processes. For AUDPC, one of the most interpretable candidates was detected on chromosome 6B, where the association mapped near TraesCS6B03G0179200 (RGA2-like disease resistance protein). Additional AUDPC loci were linked to regulatory functions, including TraesCS1B03G0311400 (a putative F-box protein) and TraesCS3B03G0289000 (Always Early 3), suggesting involvement in protein turnover and signaling regulation. A particularly notable AUDPC region was observed on 7D, where Tdurum_contig43589_340_dup2 was detected in both UAS seasons and mapped near TraesCS7D03G0417800 (PHLOEM UNLOADING MODULATOR), making this locus a stronger candidate for follow-up due to its within-location repeatability across years. For the IP, candidate genes showed greater consistency with mechanisms affecting early infection responses and symptom onset, including an LRR receptor-like kinase on chromosome 7B (TraesCS7B03G0190200) and a WRKY transcription factor on chromosome 3B (TraesCS3B03G0309700). Together, these findings suggest that variations in perception and signaling, as well as downstream transcriptional regulation, contribute to differences in symptom timing.

Although no single SNP was shared between AUDPC and IP, both traits mapped to several of the same chromosomes (notably 1B, 2A, 2B, 3B, 6B, and 7B), suggesting partial overlap at the regional level. Overall, the candidate set reinforces that AUDPC and IP are related but distinct components of quantitative resistance.

## 3. Discussion

GWAS-based analyses have provided valuable insights into the genetic basis of quantitative disease resistance in wheat; however, resistance to the spike blight pathogen *E. rostratum* has not previously been investigated using this approach. Most GWAS efforts in wheat have focused on diseases such as *Fusarium* head blight, spot blotch, and *S. tritici* blotch [17,19], leaving resistance to *E. rostratum* largely unexplored. The present study, therefore, represents the first GWAS-based analysis of wheat resistance to *E. rostratum*. 

The environment-specific MTAs observed in this study are consistent with the quantitative nature of resistance to *E. rostratum*. Several loci with relatively small effects typically govern quantitative resistance, and their expression may vary across environments [16,20]. The overall pattern observed here is also consistent with previous wheat disease-resistance studies, including reports of environment-dependent associations for spot blotch in the WAMI spring wheat panel [21]. Differences in temperature, humidity, disease pressure, and plant developmental stage may influence whether a locus reaches the GWAS significance threshold in a particular site-year. The significant genotype × environment interaction observed for both IP and AUDPC further supports this interpretation. Therefore, these MTAs should be considered environment-dependent candidate loci rather than broadly stable resistance loci. The chromosome 7D marker associated with AUDPC at UAS in both years was the most consistent association identified in this study.

Field evaluation of the WAMI spring wheat panel across two contrasting agroclimatic regions and two growing seasons revealed substantial phenotypic variation for both IP and AUDPC. The continuous distributions of these traits across environments indicate quantitative inheritance, which is consistent with resistance reported for other foliar necrotrophic pathogens of wheat [22,23]. The pronounced variation across locations and seasons further underscores the significant impact of environmental conditions on disease development and host response [20].

Correlation analysis provided insight into the biological interpretation of the two resistance-related traits. AUDPC showed moderate-to-strong positive correlations across environments, indicating relative consistency in the cumulative development of disease. In contrast, correlations between IP and AUDPC were weak and inconsistent, suggesting that early infection and subsequent disease progression represent partly distinct components of resistance. The relatively lower correlation observed for IP_UAS_2020 is likely attributable to environmental variation during the 2020 growing season, including differences in temperature, relative humidity, rainfall, and disease. Similar patterns have been reported for other wheat diseases, where incubation-related traits and disease severity capture different phases of the host–pathogen interaction [24,25]. These results support the use of both IP and AUDPC as complementary traits in GWAS of quantitative disease resistance.

Previous GWAS have identified several chromosomal regions associated with quantitative resistance to fungal diseases in wheat. For spot blotch resistance, genomic regions have been reported on chromosomes 1A, 3B, 7B, and 7D, while studies using the WAMI population identified associations on chromosomes 1B, 1D, 4A, 5A, 5B, 6A, 6B, 7A, and 7B [26,27]. Subsequent studies further supported the importance of chromosomes 3B, 5B, 6A, 6B, 7B, and 7D in spot blotch resistance [28,29,30]. Comparable multi-locus resistance architectures have also been reported for *Fusarium* head blight, *Septoria tritici* blotch, and *Septoria nodorum* blotch [31,32]. Although chromosome-level overlap does not validate the specific loci detected in the present study, these previous findings provide a broader biological context for the polygenic and environment-responsive nature of fungal disease resistance in wheat.

To biologically interpret the associations identified here, all predicted genes located within ±500 kb of the significant SNPs were surveyed, and the complete interval-level gene lists are provided in Supplementary Table S2. Putative candidates highlighted in the main text were prioritized based on physical proximity, predicted functional annotation, protein-domain or orthology information, and previously reported roles of related genes in plant defense. Among the IP-associated candidates, TraesCS7B03G0190200 encodes an LRR receptor-like kinase, a protein class involved in pathogen perception and activation of pattern-triggered immunity. The reported contribution of the related LRR/malectin receptor-like kinase LEMK1 to quantitative resistance against powdery mildew provides gene-family-level support for its biological plausibility [33]. TraesCS3B03G0309700 encodes a WRKY transcription factor, consistent with the established roles of WRKY proteins in defense-related transcriptional reprogramming. Wheat WRKY genes, including TaWRKY25 and TaWRKY70, have been implicated in fungal disease resistance and regulation of jasmonic acid, ethylene, and salicylic acid signaling pathways [34,35,36,37,38,39,40], suggesting that variation in defense signaling and transcriptional control may contribute to incubation-period differences. Additional candidates within associated intervals included an RGA2-like disease-resistance protein, a putative F-box protein, cytochrome P450s, protein kinases, transcription factors, and genes involved in protein turnover and cellular metabolism. Collectively, these gene classes are consistent with the polygenic nature of quantitative resistance, which may involve pathogen recognition, signal transduction, reactive oxygen species homeostasis, cell-wall reinforcement, and secondary metabolism [41,42,43]. However, these annotations and comparative studies provide biological support for candidate prioritization rather than direct evidence of causal involvement in resistance to *E. rostratum*.

Despite the predominance of environment-specific associations, one SNP on chromosome 7D was consistently associated with AUDPC across two consecutive seasons at UAS. D-genome associations are generally detected less frequently than those on the A and B genomes, likely due to lower genetic diversity, fewer polymorphic markers, and greater linkage disequilibrium, which can reduce statistical power [44,45]. Therefore, the smaller number of D-genome associations should not be interpreted as an absence of resistance-related loci. The repeatable 7D signal was located near TraesCS7D03G0417800, annotated as a PHLOEM UNLOADING MODULATOR-like gene, suggesting that transport- or vascular-related processes may influence cumulative disease development under the UAS environment. Overall, the distribution of MTAs across multiple chromosomes and the absence of a major-effect locus support a polygenic basis of resistance to *Exserohilum* spike blight, consistent with other wheat foliar diseases [46,47].

Although the panel was evaluated across two contrasting agroclimatic zones over two seasons, most MTAs were not consistently detected across all sites and years, indicating that loci governing IP and AUDPC are environment-dependent within the tested range. Phenotyping was conducted using a single highly virulent *E. rostratum* isolate, MTU-1001, which was selected based on previous molecular identification, pathogenicity testing, and host–pathogen interaction studies that demonstrated its high virulence against wheat [15]. The use of the same isolate across all locations and growing seasons ensured consistency in disease screening and enabled assessment of the environmental stability of resistance responses to a standardized pathogen genotype. Nevertheless, the identified associations may represent resistance responses conditioned by MTU-1001 and should not be interpreted as evidence of broad-spectrum resistance to genetically diverse *E. rostratum* populations. Future studies should incorporate isolates representing diverse levels of aggressiveness and geographic origins to quantify genotype × isolate (G × I) and genotype × environment × isolate (G × E × I) interactions and to distinguish broadly effective loci from isolate-specific responses. The identified loci should also be validated in independent association panels or biparental populations before their use in breeding. Candidate-gene nomination within a ±500 kb window provides a practical starting point; however, the mapping resolution remains limited. Therefore, key regions, particularly the repeatable chromosome 7D AUDPC-associated locus, should be refined using local linkage disequilibrium and haplotype analyses, followed by functional validation of prioritized genes using approaches such as virus-induced gene silencing or CRISPR-based targeted mutagenesis.

## 4. Materials and Methods

### 4.1. Plant Materials

The present study utilized 289 spring wheat genotypes from the Wheat Association Mapping Initiative (WAMI) panel. This panel consists of advanced elite, high-yielding lines developed for GWAS and has been widely used to dissect the genetic basis of complex agronomic and disease-related traits [48,49]. The WAMI panel was assembled through the International Wheat Improvement Network (IWIN), and seed was obtained from the Global Wheat Program of the International Maize and Wheat Improvement Center (CIMMYT), El Batán, Texcoco, Mexico.

### 4.2. Experimental Sites

The two sites differ in their temperature and humidity regimes, as well as their seasonal rainfall patterns, which influence the initiation and development of foliar diseases. BHU, Varanasi (Indo-Gangetic Plains) typically represents a north Indian wheat environment with cool winter cropping and frequent late-season warming. In contrast, UAS, Dharwad (South India), represents comparatively warmer conditions and distinct moisture dynamics during the wheat season. Evaluating genotypes across these contrasting agro-climatic zones increases the likelihood of capturing genotype × environment interactions. It strengthens confidence in loci that show consistent effects across environments, thereby improving the relevance of identified resistance sources for broad deployment.

### 4.3. Field Experimental Design and Agronomic Practices

The experiments were conducted using an alpha-lattice design with two replications in each season (2019 and 2020) at both locations. Each genotype was sown in 1 m-long rows with 25 cm row-to-row spacing and 5 cm plant-to-plant spacing. Approximately 20 seeds were manually sown per row, and an optimal plant stand was maintained by thinning excess seedlings during hand-weeding 25 days after sowing (DAS). Two rows of the early-maturing and highly susceptible cultivar ‘Sonalika’ were planted as border rows around the experimental plots to facilitate uniform disease development and serve as a spreader of inoculum. Fertilizer was applied at 120:60:40 kg ha^−1^ (N:P_2_O_5_:K_2_O). The full doses of P_2_O_5_ and K_2_O and one-third of the nitrogen were applied as a basal dose at sowing. The remaining nitrogen was applied in two equal splits: one-third at the crown root initiation stage (after the first irrigation) and one-third at anthesis (during the third irrigation). All recommended agronomic practices were followed uniformly at both locations.

### 4.4. Pathogen Isolate and Inoculum Preparation

A single highly virulent isolate of *E. rostratum* (MTU-1001) was used for artificial inoculation in all environments. The isolate was selected based on previous molecular identification and pathogenicity studies demonstrating its high virulence on wheat and suitability for resistance screening [15]. The same isolate was used across all locations and growing seasons to ensure consistency in disease evaluation. The isolate was maintained on potato dextrose agar (PDA) at 25 ± 2 °C. For inoculum production, sporulation was induced on actively growing cultures. Conidia were harvested by flooding the plates with sterile distilled water and gently dislodging spores with a sterile glass rod. The suspension was filtered through a double-layer sterile muslin to remove mycelial fragments. Spore density was determined using a haemocytometer and adjusted to 1 × 10^4^ conidia mL^−1^ with sterile distilled water. Tween-20 was added at a low concentration when necessary to improve adhesion to spikelets. The inoculum was thoroughly mixed and used immediately for spike inoculation.

### 4.5. Phenotyping for Exserohilum Spike Blight Resistance

Phenotypic screening of wheat genotypes for resistance to spike blight was conducted under field conditions at both locations. Disease assessment focused on the adult plant stage, where symptom expression is most pronounced and representative of field-level resistance. Artificial inoculation was performed using the spike inoculation technique, as described by Warham et al. [50]. Inoculation was performed at the booting stage (Zadoks growth stage GS43) [51] by injecting the spore suspension (1 × 10^4^ conidia mL^−1^) into the spike using 1 mL disposable injector syringes fitted with removable metal needles (point style 220). For each genotype, ten randomly selected plants were inoculated. The process was repeated two days after the initial application. From anthesis to the early dough stage, the field was subjected to morning mist spraying to maintain high humidity, thereby facilitating spike blight development.

For each genotype within each replication, five symptomatic spikelets were selected and scored, and the observations were averaged to obtain a representative disease estimate for that replicate. The incubation period (IP) was calculated as the number of days after inoculation to the appearance of the first visible symptom, following Parlevliet [52]. Disease severity (DS) was then quantified as the mean percentage of spikelets affected per spike and was recorded weekly from symptom initiation until the end of the season. The area under the disease progress curve (AUDPC) was computed from the sequential DS (%) values using the trapezoidal integration method described by Shaner and Finney [53]:

AUDPC=i=1∑n−1 [2(yi +yi+1 ) ×(ti+1 −ti )]

where *y_i_* and *y*_*i*+1_ represent disease severity values at the *ith* and *(*i* + 1)*th observations, *t_i_* and *t*_*i*+1_ represent the corresponding times (days after inoculation), and *n* is the total number of observations. AUDPC values were used as quantitative traits in downstream GWAS because they integrate disease development over time.

### 4.6. Genotyping

Genotypic data for the WAMI panel were obtained from the publicly available CIMMYT dataset (http://hdl.handle.net/11529/10714; accessed on 15 June 2023), in which 289 spring wheat lines were previously genotyped using the Illumina iSelect 90K SNP array (Illumina, Inc., San Diego, CA, USA), providing high-density genome-wide marker coverage in bread wheat. The raw dataset contained 18,704 polymorphic SNPs. Markers were then quality-filtered by removing SNPs with minor allele frequency (MAF) < 0.05, resulting in 15,737 high-quality SNPs for GWAS. To account for population stratification, principal component analysis (PCA) was performed using the filtered SNP dataset, and the first three principal components (PC1–PC3; PCA total = 3) were included as covariates in the GWAS model.

### 4.7. Genome-Wide Association Study

Genome-wide association analyses were performed in R version 4.5.1 using the GAPIT package, initially evaluating multiple GWAS models. Model fit and control of spurious associations were compared using quantile–quantile (QQ) plots. FarmCPU model was selected for final inference because it provided the best balance between statistical power and control of inflation/deflation [54]. The analysis used 15,737 SNP markers (MAF ≥ 0.05) and accounted for relatedness and population stratification by including a kinship (K) matrix and the first three principal components (PC1–PC3; PCA total = 3) as covariates. Marker–trait associations were declared significant based on an FDR-adjusted criterion, with a reporting threshold of −log_10_(*p*) ≥ 3.0 (*p* ≤ 0.001) applied, consistent with previous GWAS conducted using the WAMI panel [49,55]. Manhattan and QQ plots were generated to visualize associations and further assess model performance.

### 4.8. Candidate Gene Identification

Significant SNPs were annotated against the bread wheat reference genome IWGSC CS RefSeq v2.1 (IWGSC Annotation v2.1) to determine their physical positions and identify nearby candidate genes. Candidate genes were prioritized by interrogating genomic intervals surrounding each significant SNP (typically ±500 kb, following common practice in wheat GWAS, where candidate selection is guided primarily by physical proximity and previously reported LD-based conventions, rather than estimating LD for every locus) using Ensembl Plants (https://plants.ensembl.org; accessed on 29 January 2026) and the associated gene functional descriptions. When wheat gene annotations were limited, orthology-based information from related plant species was used to support functional inference. Sequences of the nominated putative candidate genes were further examined using NCBI BLASTn (version 2.17.0+; https://blast.ncbi.nlm.nih.gov/Blast.cgi; accessed on 15 February 2026), and available protein domain annotations and expression information were retrieved when accessible.

### 4.9. Statistical Analyses

All statistical analyses were performed in R version 4.5.1. Phenotypic data for IP and AUDPC were analyzed using analysis of variance (ANOVA) to test for significant differences among genotypes, with location and season considered in the model where appropriate for multi-environment evaluation. GWAS were conducted using the GAPIT package, with an initial assessment of multiple GWAS models. Model adequacy and control of spurious associations were evaluated using quantile–quantile (QQ) plots. FarmCPU was selected for final analysis based on the best fit and genomic inflation control. Association mapping used 15,737 SNPs (MAF ≥ 0.05) and accounted for relatedness and population structure by including the kinship (K) matrix and the first three principal components (PC1–PC3) as covariates. Marker–trait associations were declared significant using an FDR-adjusted criterion, and for reporting and visualization, a threshold of −log_10_(*p*) ≥ 3.0 (*p* ≤ 0.001) was applied. Genotype stability across the four environments was assessed using the AMMI model. AMMI stability values were calculated from IPCA1 and IPCA2 scores, with lower values indicating greater stability. Genotypes with AUDPC in the lowest quartile and IP in the highest quartile were considered favorable and ranked using their combined AUDPC and IP stability ranks.

## 5. Conclusions

This study provides the first GWAS-based assessment of wheat resistance to *E. rostratum* associated spike blight using an elite association panel evaluated across two Indian agroclimatic zones over two seasons. The continuous variation observed for incubation period and AUDPC supports a quantitative, polygenic basis of resistance, with these traits capturing partly distinct components of disease response. GWAS identified multiple significant marker–trait associations, including a repeatable AUDPC locus on chromosome 7D, and candidate gene mining highlighted biologically plausible genomic regions for further investigation. These results establish a genetic framework for multi-isolate validation, functional characterization, and development of breeder-friendly markers to facilitate deployment of resistance in wheat improvement programs.

## Figures and Tables

**Table 1:** Analysis of variance for IP and AUDPC.

Source	df	IP (F Value)	IP (*p* Value)	AUDPC (F Value)	AUDPC (*p* Value)
Genotype (G)	288	4.9	<0.001 *	7.2	<0.001 *
Environment (E)	3	58.2	<0.001 *	120.5	<0.001 *
G × E	864	1.6	<0.001 *	1.9	<0.001 *
Error	1156	—	—	—	—

df = Degrees of freedom, * Significant *p* < 0.001.

**Table 2:** Mean disease response and AMMI stability values of the highest-ranked wheat genotypes across four environments.

Genotype	Mean AUDPC	AUDPC ASV	Mean IP (Days)	IP ASV	Overall Stability Rank
3868699	429.63	1.271	4.25	0.174	1
1370653	376.25	1.972	3.88	0.179	2
346095	378.88	0.887	4	0.544	3
1403557	380.63	2.393	3.88	0.179	4
346479	365.75	0.928	3.88	0.58	5
5551787	388.5	2.877	4.25	0.038	5

Note—Lower AUDPC values indicate greater resistance, whereas longer incubation periods indicate delayed disease development. Lower ASVs indicates greater stability across environments. Genotypes were considered phenotypically favorable when their mean AUDPC values were in the lowest quartile and their mean IP values were in the highest quartile. Overall stability ranks were based on the summed ranks of AUDPC and IP ASV among the 26 genotypes that met both phenotype criteria. Genotypes 346479 and 5551787 shared a stability rank of 5.

**Table 3:** List of significant GWAS SNPs and annotated candidate genes associated with IP and AUDPC across BHU and UAS environments (2019–2020).

SNP	Chr ^1^	Pos cM ^2^	Trait ^3^	Location	Year	Candidate Gene ID ^4^	Putative Candidate Gene Description ^5^
RAC875_c17011_373	6B	122	AUDPC	BHU	2019	TraesCS6B03G0303300	Anthranilate phosphoribosyltransferase
BobWhite_c9424_243	3B	110	AUDPC	BHU	2019	TraesCS3B03G0289000	Always early 3
Tdurum_contig61914_169	1B	141	AUDPC	BHU	2019	TraesCS1B03G0311400	Putative F-box protein
RAC875_c64292_194	6B	57	AUDPC	BHU	2020	TraesCS6B03G0179200	Disease resistance protein RGA2
Tdurum_contig892_633	1B	78	AUDPC	BHU	2020	TraesCS1B03G0220100	Uncharacterized chloroplastic-like protein
Tdurum_contig10579_790	2A	105	AUDPC	BHU	2020	TraesCS2A03G0313000	Pentatricopeptide repeat-containing protein
tplb0042o21_419	2B	160	AUDPC	UAS	2019	TraesCS2B03G0430300	Probable transcription factor
Excalibur_c16687_476	7B	73	AUDPC	UAS	2019	TraesCS7B03G0175900	Nucleolar GTP-binding protein 1
TA015732-1144	5B	25	AUDPC	UAS	2019	TraesCS5B03G0061000	Serine protease HTRA3-like
Tdurum_contig43589_340_dup2	7D	141	AUDPC	UAS	2019	TraesCS7D03G0417800	Phloem unloading modulator
wsnp_Ex_c57322_59084809	3A	80	AUDPC	UAS	2019	TraesCS3A03G0244400	Phosducin-like protein 3
BobWhite_c42974_184	7B	73	AUDPC	UAS	2020	TraesCS7B03G0175800	Uncharacterized protein
Tdurum_contig43589_340_dup2	7D	141	AUDPC	UAS	2020	TraesCS7D03G0417800	Phloem unloading modulator
TA002702-0616	3A	117	IP	BHU	2019	TraesCS3A03G0306200	Protodermal factor 1
BobWhite_c15763_205	3B	124	IP	BHU	2019	TraesCS3B03G0309700	WRKY transcription factor 72
RAC875_c2254_1169	6B	120	IP	BHU	2020	TraesCS6B03G0297800	UBX domain-containing protein 4-like
Tdurum_contig19852_242	7B	78	IP	UAS	2019	TraesCS7B03G0190200	LRR receptor-like serine/threonine-protein kinase ER1
wsnp_Ku_c33287_42805533	2B	96	IP	UAS	2019	TraesCS2B03G0294800	Mediator of RNA polymerase II transcription subunit 33A-like
BobWhite_rep_c49916_325	7A	56	IP	UAS	2019	TraesCS7A03G0211000	ATP-dependent 6-phosphofructokinase 5, chloroplastic-like
BS00023031_51	4A	61	IP	UAS	2020	TraesCS4A03G0127900	Chaperone protein DnaJ-like
Excalibur_c8998_469	3B	80	IP	UAS	2020	TraesCS3B03G0234800	Glyoxysomal fatty acid beta-oxidation multifunctional protein MFP-a-like
BS00022372_51	6A	126	IP	UAS	2020	TraesCS6A03G0346600	Protein RGF1 inducible transcription factor 1
TA001874-1495	2B	53	IP	UAS	2020	TraesCS2B03G0188400	Anthocyanidin 5,3-O-glucosyltransferase
IAAV2376	2A	101	IP	UAS	2020	TraesCS2A03G0308200	Novel plant SNARE 13
Excalibur_c25640_110	1B	173	IP	UAS	2020	TraesCS1B03G0359800	Uncharacterized LOC123114204

Candidate genes were identified by anchoring significant SNP markers to the IWGSC Chinese Spring RefSeq v2.1 wheat reference genome and surveying genes within ±500 kb of each marker. Functional annotations were obtained from Ensembl Plants, NCBI BLAST v 2.17.0, and available orthology/domain information. ^1^ Chr = chromosome; ^2^ Pos cM = genetic position in centimorgans; ^3^ Trait = disease-related phenotype associated with the marker, AUDPC = area under the disease progress curve; IP = incubation period; ^4^ Candidate Gene ID = annotated wheat gene identifier located near the significant SNP; ^5^ Candidate Gene Description = predicted or annotated function of the candidate gene based on available genome annotation and sequence similarity.

## Data Availability

The raw data supporting the conclusions of this article will be made available by the authors on request.

## Supplementary Materials

The following supporting information can be downloaded at: https://www.mdpi.com/article/10.3390/plants15152351/s1. Figure S1: Q-Q Plots for AUDPC and IP; Table S1: Mean AUDPC, mean IP, AMMI stability values, trait-specific stability ranks, combined rank scores, and overall stability ranks of the 26 wheat genotypes meeting both phenotypic selection criteria across four environments. Table S2: Predicted candidate genes identified within ±500 kb of significant SNP markers associated with incubation period and AUDPC for *Exserohilum* spike blight resistance in the WAMI wheat panel.

## Abbreviations

The following abbreviations are used in this manuscript:

WAMI	Wheat Association Mapping Initiative
IP	Incubation Period
AUDPC	Area Under the Disease Progress Curve
GWAS	Genome-Wide Association Study
SNP	Single Nucleotide Polymorphism
MAF	Minor Allele Frequency
MTA	Marker Trait Association
ANOVA	Analysis of Variance
GAPIT	Genome Association and Prediction Integrated Tool
FarmCPU	Fixed and Random Circulating Probability Unification
LRR	Leucine-Rich Repeat
